# p63 suppresses the ability of pregnancy-identified mammary epithelial cells (PIMECs) to drive HER2-positive breast cancer

**DOI:** 10.1038/s41419-021-03795-5

**Published:** 2021-05-22

**Authors:** Christopher E. Eyermann, Jinyu Li, Evguenia M. Alexandrova

**Affiliations:** Department of Pathology and Stony Brook Cancer Center, Stony Brook University, Stony Brook, NY 11794-8691 USA

**Keywords:** Breast cancer, Mechanisms of disease

## Abstract

While pregnancy is known to reduce a woman’s life-long risk of breast cancer, clinical data suggest that it can specifically promote HER2 (human EGF receptor 2)-positive breast cancer subtype (HER2+ BC). HER2+ BC, characterized by amplification of HER2, comprises about 20% of all sporadic breast cancers and is more aggressive than hormone receptor-positive breast cancer (the majority of cases). Consistently with human data, pregnancy strongly promotes HER2+ BC in genetic mouse models. One proposed mechanism of this is post-pregnancy accumulation of PIMECs (pregnancy-identified mammary epithelial cells), tumor-initiating cells for HER2+ BC in mice. We previously showed that p63, a homologue of the tumor suppressor p53, is required to maintain the post-pregnancy number of PIMECs and thereby promotes HER2+ BC. Here we set to test whether p63 also affects the intrinsic tumorigenic properties of PIMECs. To this end, we FACS-sorted YFP-labeled PIMECs from p63+/−;ErbB2 and control p63+/+;ErbB2 females and injected their equal amounts into immunodeficient recipients. To our surprise, p63+/− PIMECs showed increased, rather than decreased, tumorigenic capacity in vivo, i.e., significantly accelerated tumor onset and tumor growth, as well as increased self-renewal in mammosphere assays and proliferation in vitro and in vivo. The underlying mechanism of these phenotypes seems to be a specific reduction of the tumor suppressor TAp63 isoform in p63+/− luminal cells, including PIMECs, with concomitant aberrant upregulation of the oncogenic ΔNp63 isoform, as determined by qRT-PCR and scRNA-seq analyses. In addition, scRNA-seq revealed upregulation of several cancer-associated (Il-4/Il-13, Hsf1/HSP), oncogenic (TGFβ, NGF, FGF, MAPK) and self-renewal (Wnt, Notch) pathways in p63+/−;ErbB2 luminal cells and PIMECs per se. Altogether, these data reveal a complex role of p63 in PIMECs and pregnancy-associated HER2+ BC: maintaining the amount of PIMECs while suppressing their intrinsic tumorigenic capacity.

## Introduction

HER2 (human epidermal growth factor receptor 2)-positive breast cancer (HER2+ BC) comprises 15–20% of all sporadic breast cancers and is an aggressive subtype. It is characterized by gene amplification/protein upregulation of HER2 receptor tyrosine kinase and presents with reduced survival and high rate of relapse after chemotherapy, due to enhanced cell proliferation, angiogenesis, metastasis, and reduced apoptosis^1^. HER2+ BC is frequently diagnosed at widely metastatic stage III/IV and in younger patients^2^. Although HER2-targeted therapies (Trastuzumab, Lapatinib) have greatly improved management of this malignancy, there is a significant rate of primary and acquired resistance^3,4^, urging to identify additional factors that contribute to HER2+ BC pathogenesis and survivorship.

In contrast to other subtypes, HER2+ BC seems to be associated with pregnancy. Thus, the incidence of HER2+ BC among so called pregnancy-associated breast cancers is increased to 28–58%, compared to 16–22% of age-matched control patients or 19% in the general population of reproductive age^5–10^. Moreover, parity can increase the life-long risk of HER2+ BC. Indeed, two large studies (2710 and 28,095 patients, respectively) found a significant association of parity with the risk of HER2+ BC, whereas ≥3 full-term pregnancies had an even greater association^11,12^. In agreement, parity accelerates tumor onset and mortality in two genetic mouse models of HER2+ BC, the ErbB2 and Neu mice (with constitutively active and amplified HER2, respectively)^13–15^. Compelling explanation for this came from mouse studies showing that a major cancer stem cell/tumor-initiating cell population for HER2+ BC are PIMECs (pregnancy-identified mammary epithelial cells)^13,16^. PIMECs are multipotent alveolar progenitors that comprise 0.8–4% of mammary epithelial cells (MECs) in virgins, but undergo enormous expansion in late pregnancy to give rise to essentially all milk-producing alveoli^17–19^. Importantly, PIMECs—unlike the rest of MECs—are largely resistant to apoptosis during post-lactation gland involution and now comprise 20–30% luminal cells and greatly contribute to gland expansion in subsequent pregnancies^17,19–21^. This significant increase in PIMECs content in parous females is likely the basis for their increased susceptibility to HER2 tumorigenesis. Of note, even in virgin mice, all HER2-driven tumors arise from lineage-traced PIMECs, highlighting their bone fide role as tumor-initiating cells for HER2+ BC, at least in mice^13,16^.

We previously showed that p63, an epithelial master regulator and a homologue of the tumor suppressor p53, is a critical novel regulator of PIMECs and pregnancy-associated HER2+ BC^15^. Specifically, mammary glands from heterozygous p63+/− females (homozygous p63−/− animals die perinatally^22,23^) exhibit enhanced apoptosis in post-lactation gland involution, mediated by Oncostatin M/Stat3 and reduced Neuregulin/Stat5 signaling^15^. Moreover, the post-involution p63+/− mammary glands contain on average 40% fewer PIMECs than p63+/+ glands^15^, suggesting that p63 is required to maintain the PIMECs pool. Consistently, p63+/−;ErbB2 females are partially protected from HER2+ BC (which is not observed in virgins), pointing to the reduced PIMEC content as the likely underlying mechanism^15^. Since p63 is a known regulator of normal epithelial stem cells and cancer stem cells^24–28^, here we set to test whether, besides the PIMECs content, p63 also regulates their intrinsic tumorigenic properties. Surprisingly, we found that p63 does play a role, but as a tumor suppressor rather than an oncogene. This seems to be due to an interplay between p63 isoforms: tumor suppressor TAp63 and oncogenic ΔNp63.

## Materials and methods

### Animals and mammary fat pad transplantation assay

p63+/− mice^15,23^ were a gift from Frank McKeon. Rosa-LSL-YFP mice^17^ and MMTV-ErbB2 mice^15,29^ were from the Jackson Laboratory, strains Gt(ROSA)26Sortm1(Smo/EYFP)Amc/J and FVB-Tg(MMTV-Erbb2)NK1Mul/J, respectively. WAP-Cre mice^15^ were from the NCI Mouse Repository (https://frederick.cancer.gov/science/technology/mouserepository), strain 01XA8. The littermate experimental p63+/−;Rosa-LSL-YFP;WAP-Cre;ErbB2 and control p63+/−;Rosa-LSL-YFP;WAP-Cre;ErbB2 females (“p63+/−” and “p63+/+”, respectively) on a mixed 129SVJ/C57Bl6J:FVBN (50:50) background were generated as previously described^15^, impregnated at 3 months of age and allowed to nurse pups for 10 days to stimulate induction and YFP labeling of PIMECs^17,21^. For fat pad transplantation, FACS-sorted p63+/+ and p63+/− PIMECs in 100 µl 50:50 DMEM:Matrigel (Cat # 356234, Corning) were injected into mammary glands #4 and #9 of 4–5 weeks old virgin immuno-deficient female recipients, strain Foxn1^Nu/Nu^ (the Jackson Laboratories), at ~5000 cells per site (4920 ± 594 and 5067 ± 847 of p63+/+ and p63+/− cells, respectively). The recipients were monitored weekly for tumor onset (when p63+/+ and p63+/− allografts measured 7.7 ± 1.3 mm^3^ and 8.8 ± 3.8 mm^3^, respectively, mean ± SD) and their tumors were measured weekly by caliper. Tumor volume was calculated as l*w*h/2, where “l” is length, “w” is width, “h” is height (an approximate formula for the ellipsoid volume). Allografts that did not reach 5 mm^3^ by week 12 were excluded from the analysis. All animals were treated humanely and according to the guidelines by the Stony Brook University Institutional Animal Care and Use Committee (IACUC; protocol number 924666). Males were excluded from the analysis. The sample size was not pre-determined.

### Fluorescence-activated cell sorting (FACS) and quantitative RT-PCR (qRT-PCR)

**Fig. 1:**
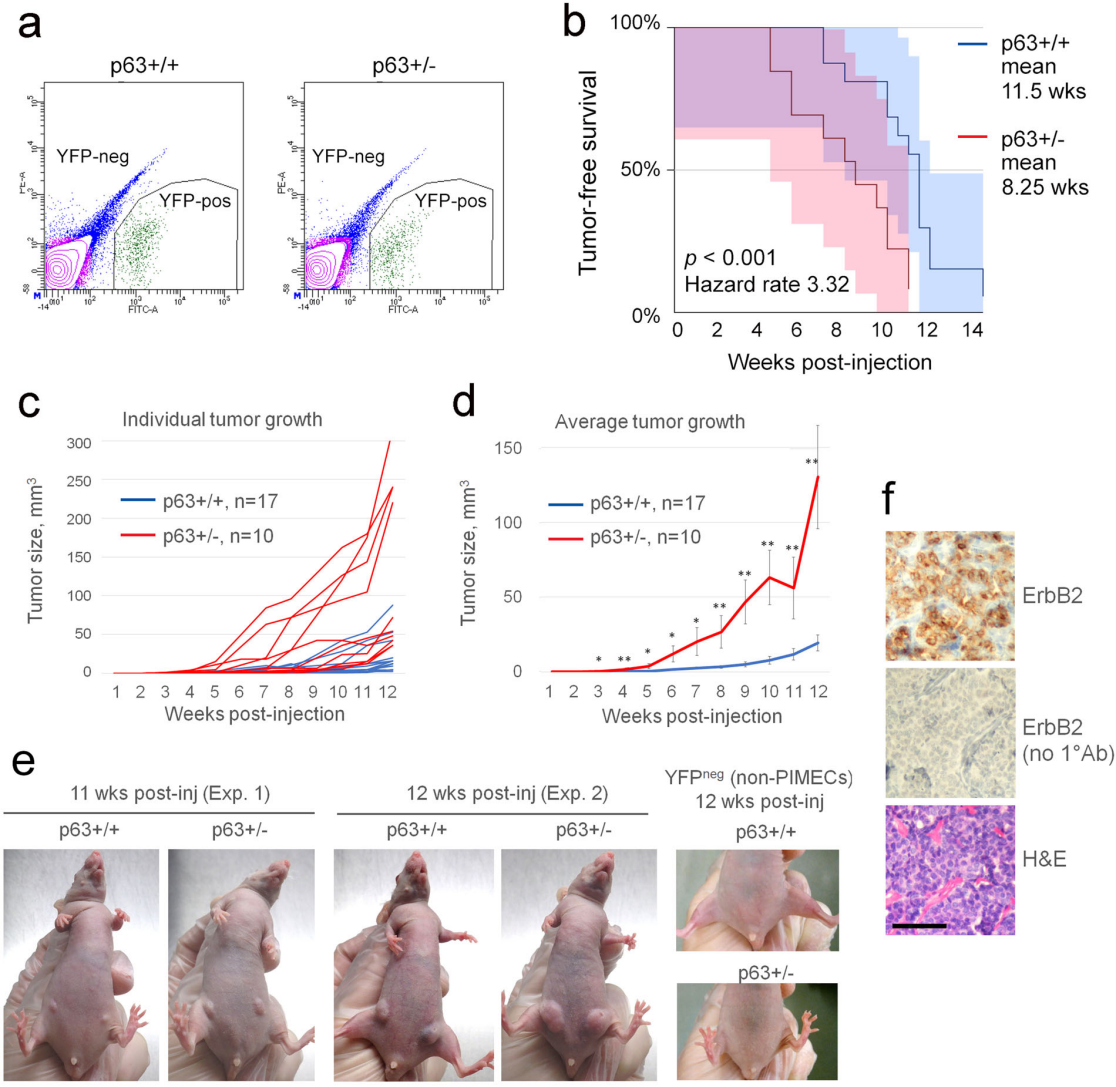
Increased tumorigenicity of p63+/− PIMECs in vivo. **a** Representative FACS sorting diagrams. **b** Tumor-free survival of the immunodeficient recipients allografted with 5000 p63+/+ (*n* = 15) or p63+/− (*n* = 12) PIMECs, Kaplan–Meier analysis, log rank statistics (*p*), shaded area is 95% confidence interval. **c** Growth of individual allografts. Note, PIMECs were obtained from four individual p63+/+ and three individual p63+/− donors and used for *n* = 17 and *n* = 10 allografts, respectively. **d** Average allograft growth, mean ± SEM, *p < 0.05, **p < 0.01. **e** Representative imag**e**s from two independent experiments. **f** PIMECs-derived tumors maintain their HER2 positivity until the endpoint, immunohistochemistry; scale bar, 100 µm.

For PIMECs isolation, p63+/− and p63+/+ females were euthanized at 6 weeks post-lactation (i.e., complete gland involution^15^), and their total MECs were isolated from mammary glands #2–5 and #7–10 as previously described^30^, immediately followed by sterile FACS sorting for YFP-positive cells, i.e., PIMECs (Fig. 1a). Freshly isolated PIMECs were used for mammary fat pad transplantation, mammosphere assays, or plated for in vitro proliferation assay. For qRT-PCR, total MECs were FACS-sorted with CD24 antibody (BD Cell Analysis, Cat #561079), and CD24^high^ (i.e., luminal) cells were used for qRT-PCR as previously described^2^ with the following primers: TAp63: ATGAATTTTGAAACTTCACGGTGTG (F), GGGTCACTGAGGTCTGAGTCTTG (R); ΔNp63: GTTGTACCTGGAAAACAATGC (F), CAGGCATGGCACGGATAAC (R). For scRNA-seq analysis, CD24^pos^ (i.e., epithelial) cells were used, see below.

### 3D floating mammosphere assay

For mammosphere assays, freshly isolated p63+/+ and p63+/− PIMECs (single cells) were plated into ultra-low-adherent 12-well plates at 6500–17,500 cells per well (see Supplementary Table 1 for details) in serum-free mammary epithelial basal cell growth medium (Lonza), supplemented with B27 (Fisher Scientific), 20 ng/ml HB-EGF, 20 ng/ml bFGF and 4 µg/ml Heparin (all from Sigma). Floating mammospheres were counted 12 days later. The mammosphere formation efficiency (MFE) was calculated as m/p*100%, where “m” is the number of mammospheres and “p” is the number of plated cells.

### In vitro and in vivo proliferation assays

For in vitro proliferation assay, freshly isolated p63+/+ and p63+/− PIMECs were plated into 24-well plates at 30,000–120,000 cells per well in DMEM/F12 media (Gibco), switched to CNT Prime media (Cellntec) after two days, and stained for the proliferation marker Ki67 either after 6 days (when 70,000–120,000 were plated) or after 12 days (when 30,000 cells were plated), as described below. Five random non-overlapping fields were photographed at ×20 magnification, and the percent of Ki67-positive cells was calculated. For the in vivo proliferation assay, mammary gland #4 from p63+/+ and p63+/− sisters were flattened on filter paper, fixed in formalin, embedded in paraffin, and sectioned (5 μm), followed by immunofluorescent staining as described below. Ten random non-overlapping fields were photographed at ×20 magnification, and the percent of Ki67-positive cells within YFP-positive cells was calculated.

### Immunofluorescence

For immunofluorescent staining of proliferating PIMECs, cells were fixed with 4% paraformaldehyde, blocked in 5% goat serum, and incubated with primary antibodies (Ki67, 1:200, Cell Signaling, Cat #12202; GFP, 1:200, Aves Labs, Cat #GFP-1020) overnight at 4 °C. After PBS washing, slides were incubated with secondary antibodies (goat anti-rabbit, Alexa Fluor 594, Invitrogen, Cat #A-11012; goat anti-chicken, Alexa Fluor 488, Invitrogen, Cat #A-11039), followed by counterstain with DAPI. For immunofluorescent staining of mammary glands, slides were deparaffinized, boiled in citrate antigen retrieval buffer (Vector Labs) for 15 min, blocked in 5% goat serum, and incubated with primary antibodies (Ki67, 1:400, Cell Signaling, Cat #12202; GFP in lieu of YFP, 1:400, Aves Labs, Cat #GFP-1020) overnight at 4 °C. After PBS washing, slides were incubated with fluorescent secondary antibodies (as above), or (Fig. 1f) biotinylated secondary antibody (Invitrogen, Cat #31820) followed by Vectastain ABC-HRP (Vector Labs, Cat #7200) and DAB Quanto substrate (Thermo Fisher Scientific) with hematoxylin counterstain (or hematoxylin and eosin). Coverslips were mounted with Prolong Gold with DAPI (Invitrogen). Images were taken with Nikon Eclipse Ti-S microscope (Nikon) using NIS-Elements AR software (Nikon).

### Single-cell RNA sequencing (scRNA-seq)

Cell suspensions of p63+/+ and p63+/− PIMECs obtained as described above from four pooled p63+/+ or three pooled p63+/− mice, respectively, were loaded on a 10x Genomics Chromium instrument to generate single-cell gel beads in emulsion (GEMs). Approximately 45,000 cells of each genotype were loaded per channel. ScRNA-seq libraries were prepared using the following kits: Chromium Next GEM Single Cell 3′ GEM, Library & Gel Bead Kit v3.1, PN-1000121; Chromium Next GEM Chip G Single Cell Kit, PN-1000120 and Single Index Kit T Set A PN-1000213 (10x Genomics) as described^31^ and following the User Guide (manual part #CG000204 Rev D). Libraries were run on an Illumina NovaSeq 6000 paired-end reads, read 1 is 28 cycles, i7 index is 8 cycles, and read 2 is 91 cycles, one lane per sample, for approximately >57% and >48% sequencing saturation (p63+/+ and p63+/−, respectively). The Cell Ranger Single Cell Software Suite (v1.3) was used to perform UMI processing and single-cell 3′ gene counting. Cell Ranger (v5.0.1), Loupe Browser (v5.0.0), and R package Seurat (v4.0.0) were used to visualize gene expression and find significantly altered genes. For best cell type identification and separation, we chose K-mean clustering (*k* = 8) and UMAP for dimensionality reduction. Gene expression analysis of luminal cells (Figs. 3b–e, 4, 5a) was performed using Loupe browser and the Significant Feature Comparison/Locally Distinguishing function. Gene expression analysis of PIMEC cells (Fig. 5b–e) was performed using Seurat, where exogenous *YFP* gene was added to identify PIMECs. The control and experimental samples were processed with “cellranger count”, followed by re-aggregation into a combined dataset using “cellranger aggr”. Again, UMAP was used (resolution = 0.015), and differentially expressed genes between p63+/− and p63+/+ PIMECs were found by comparing YFP/Krt8 double-positive cells (expression > 0), using FindMarkers function. Wilcoxon rank-sum test and Bonferroni corrections were used to calculate adjusted *p*-values.

### Statistical analysis

Mouse tumor-free survival was analyzed by Kaplan–Meier analysis and log rank statistics, and the *p* value and the hazard rate were determined using online software (https://www.evanmiller.org/ab-testing/survival-curves.html). Tumor size and the mammosphere formation efficiency were analyzed by unpaired two-tailed Student’s *t*-test, *p* < 0.05 was considered statistically significant. No animal randomization was used. No blinding was used. No statistical method was used to pre-determine sample size. Normal distribution of data and data variation was not assessed.

## Results

### Accelerated HER2 tumorigenesis of p63+/− PIMECs

To test whether p63 regulates the intrinsic tumorigenic property of PIMECs, we sought to isolate PIMECs from p63+/+ and p63+/− females and transplant their equal amounts into immunodeficient recipients via mammary fat pad transplantation^20^. As cancer stem cells, HER2-expressing PIMECs give rise to mammary tumors in this assay^32^. To this end, we generated p63+/−;WAP-Cre;Rosa-LSL-YFP;ErbB2 and control p63+/+;WAP-Cre;Rosa-LSL-YFP;ErbB2 cohorts (hereafter “p63+/−” and “p63+/+”, respectively), which are similar to our previously described model^15^, except for in vivo YFP lineage tracing instead of in vitro detectible LacZ^17,19,21^. In these mice, the Cre recombinase driven by the WAP (whey acidic protein) promoter is activated in the second half of pregnancy and efficiently removes the LoxP-Stop-LoxP (LSL) cassette upstream of YFP, thus permanently labeling PIMECs and their stable post-lactation progeny^17–21,33^. Freshly FACS-isolated p63+/+ and p63+/− PIMECs (Fig. 1a) were injected at equal amounts (about 5000 cells per site) into mammary glands #4 and #9 of immunodeficient female recipients. To our surprise—and contrary to our expectations—p63+/− allografts appeared much earlier than p63+/+ allografts in all three independent experiments. Thus, tumor-free survival was 1.4 times shorter for p63+/− PIMECs, median 8.25 weeks vs. 11.5 weeks in controls (Fig. 1b), and the growth rate was significantly faster for p63+/− allografts (Fig. 1c–f). Of note, YFP-negative (non-PIMEC) MECs did not produce tumors, confirming that PIMECs are true tumor-initiating cells (Fig. 1e, right).

### Increased self-renewal and proliferation capacity of p63+/− PIMECs

**Fig. 2:**
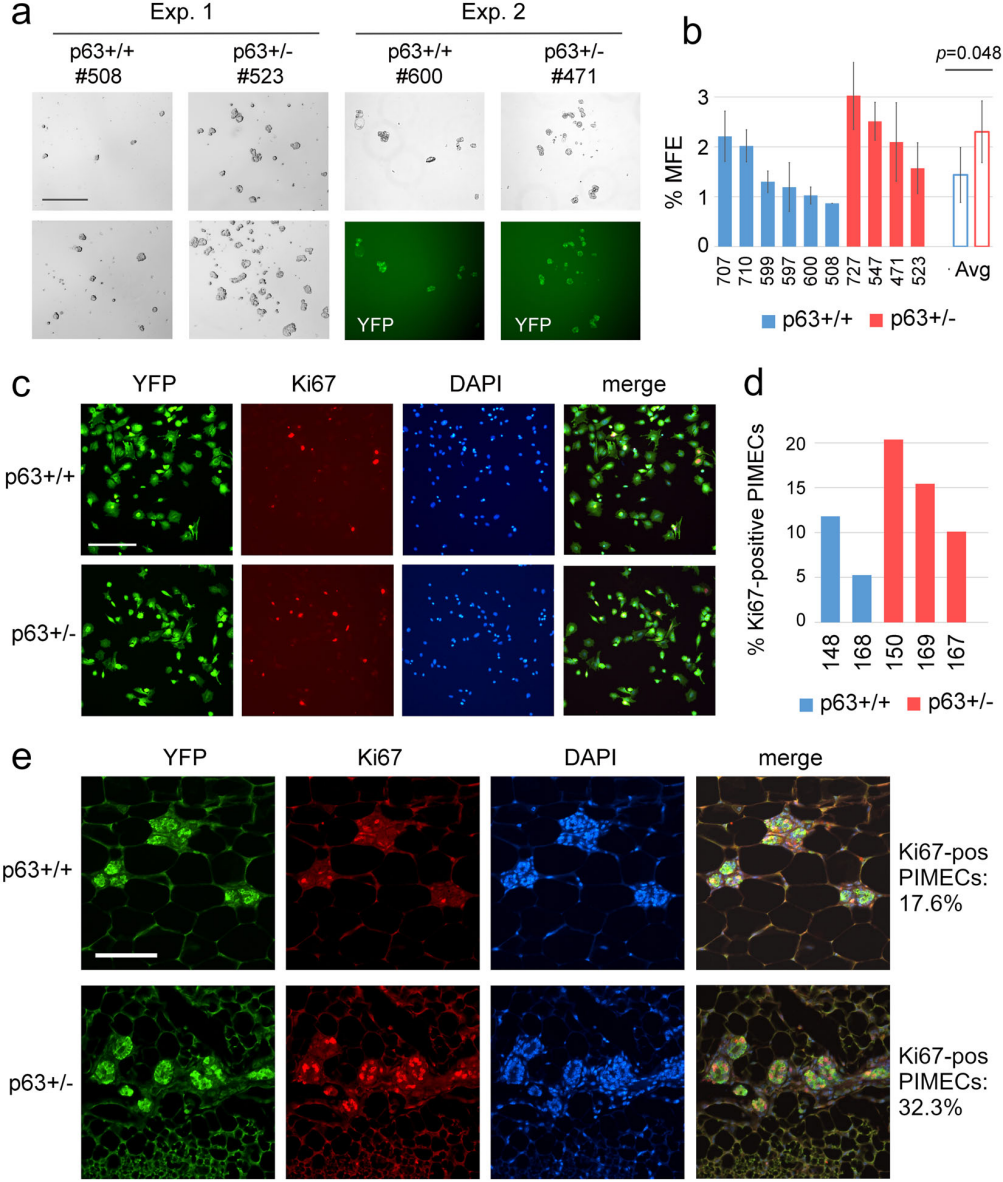
Increased self-renewal and proliferation of p63+/− PIMECs. **a**, **b** Floating mammosphere assay. **a** Two representative experiments out of three in total. ~15,000 PIMECs (Exp. 1, two different fields are shown) and ~10,000 PIMECs (Exp. 2) were seeded in 12-well plates and analyzed 12 days later. YFP, PIMECs auto-fluorescence. Scale bar, 250 µm. **b** Summary of three independent experiments, open bars are mean ± SD of all independent p63+/+ (*n* = 6) and p63+/− (*n* = 4) PIMEC isolates, respectively. MFE, mammosphere formation efficiency. See Supplementary Table 1 for more details. **c**, **d** PIMECs proliferation in vitro. Freshly FACS-sorted p63+/+ (*n* = 2) and p63+/− (*n* = 3) PIMECs were plated onto adherent 24-well plates, followed by immunofluorescent staining for YFP (i.e., PIMECs) and Ki67. **c** Representative images; scale bar, 100 µm. **d** Quantification. **e** PIMECs proliferation in vivo. Mammary glands from a single p63+/+ and p63+/− sister pair were dissected at 6 weeks post-lactation (the complete involution stage) and stained for YFP and Ki67. Representative images; scale bar, 100 µm.

Cancer stem cells are characterized by the ability to give rise to the bulk of the tumor and—similarly to normal stem/progenitor cells—to self-renew and give rise to floating spheres in suspension conditions^20,26^. We hypothesized that the more aggressive nature of p63+/− PIMECs in transplantation assays may be due to their increased self-renewal capacity. To test this, we used the 3D floating mammosphere forming assay, which is widely used to test stem cell activity in tissues, tumors, and cell lines^34^. Spheroids originate from rare cells with stem cell features able to grow in suspension and behaving as tumorigenic in mice^34^. To this end, we plated freshly isolated single-cell suspensions of p63+/+ and p63+/− PIMECs onto ultra-low-adherent 12-well plates (6500–17,500 cells per well, see Supplementary Table 1). This induced formation of mammospheres within 7–12 days, as previously reported^21^. As expected, the mammosphere formation efficiency (MFE) was significantly higher for p63+/− than p63+/+ PIMECs: 2.30 ± 0.62 vs. 1.44 ± 0.55, respectively (Fig. 2a, b). The average mammosphere size was not different between the genotypes (data not shown).

As another possible mechanism of the increased tumorigenic capacity of p63+/− PIMECs, we then assessed their proliferation potential in vitro and in vivo. To this end, we plated freshly isolated p63+/+ and p63+/− PIMECs onto adherent 24-well plates and, 6–12 days later, stained them for the cell proliferation marker Ki67, frequently used in the clinic to assess tumor aggressiveness. As expected, p63+/− PIMECs had on average 1.8-folds higher proliferation index than p63+/+ PIMECs (Fig. 2c, d). We also attempted to analyze Ki67 in the PIMECs of unperturbed mammary glands, i.e., in vivo, but found it problematic, likely due to high-fat content, as previously reported^35^. Nevertheless, a single p63+/+ and p63+/− sister pair again revealed a 1.8-fold higher PIMECs proliferation rate in the p63+/− compared to p63+/+ gland (Fig. 2e). In sum, these data uncovered a novel tumor suppressor—rather than oncogenic—role of p63 in PIMECs, which is associated with altered self-renewal and proliferation ability.

### Downregulation of TAp63 and upregulation of ΔNp63 in p63+/− luminal cells and PIMECs

**Fig. 3:**
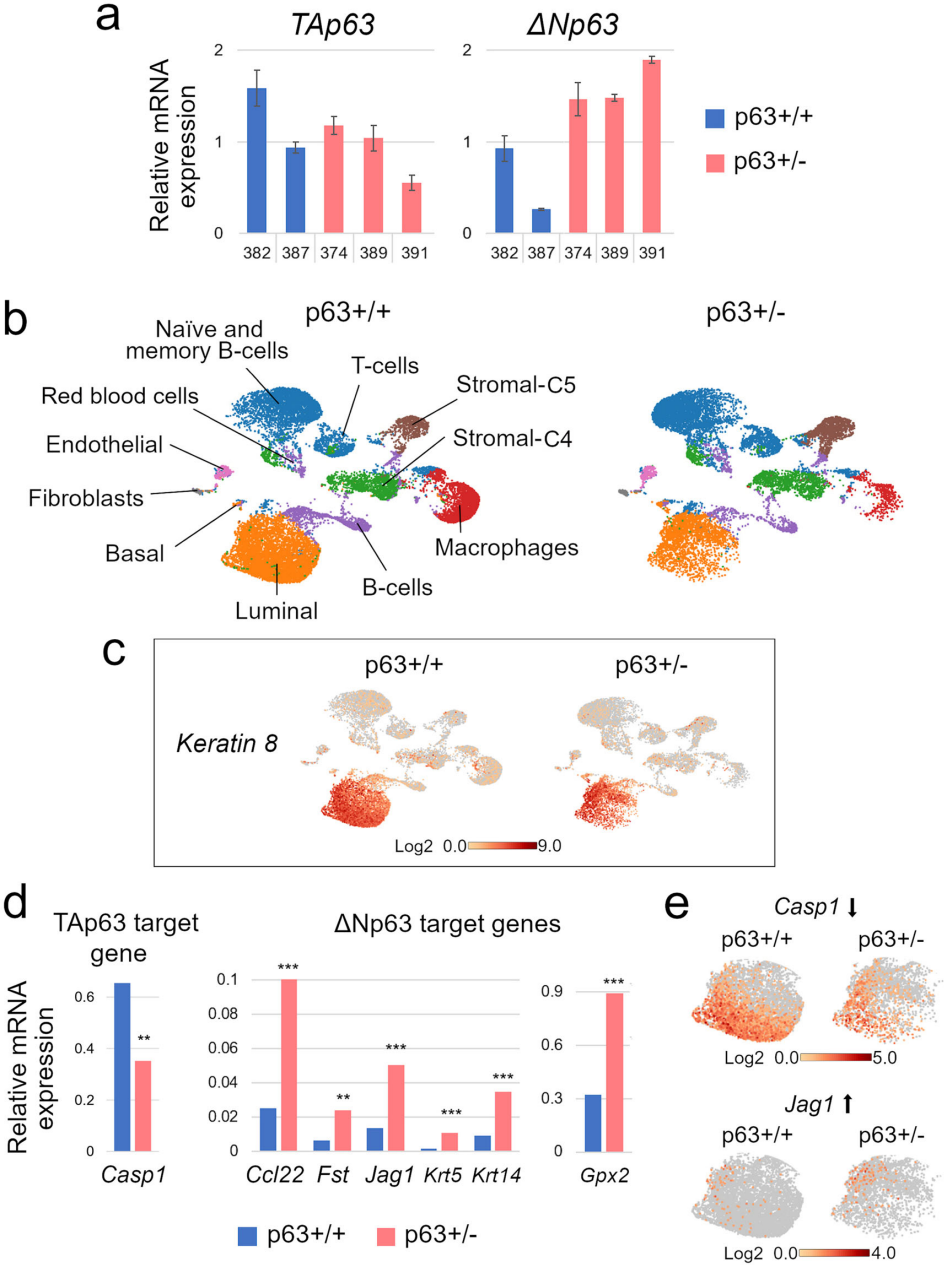
Downregulation of TAp63 and upregulation of ΔNp63 isoforms in p63+/− luminal cells. **a** Quantitative RT-PCR on independent p63+/+ (*n* = 2) and p63+/− (*n* = 3) CD24^high^ (i.e., luminal) FACS-sorted cells. **b**–**e** ScRNA-seq analysis of pooled p63+/+ (*n* = 4) and p63+/− (*n* = 3) CD24^pos^ (i.e., mammary epithelial) cells. **b** UMAP plots visualized in Loupe browser, see Supplementary Table 2 for details. **c** *Krt8* marks luminal cells. **d**, **e** Average expression (**d**) and representative UMAP plots (**e**) of significantly altered TAp63 and ΔNp63 target genes in the luminal cluster of p63+/+ and p63+/− mammary epithelial cells, **p < 0.01, ***p < 0.001.

**Fig. 4:**
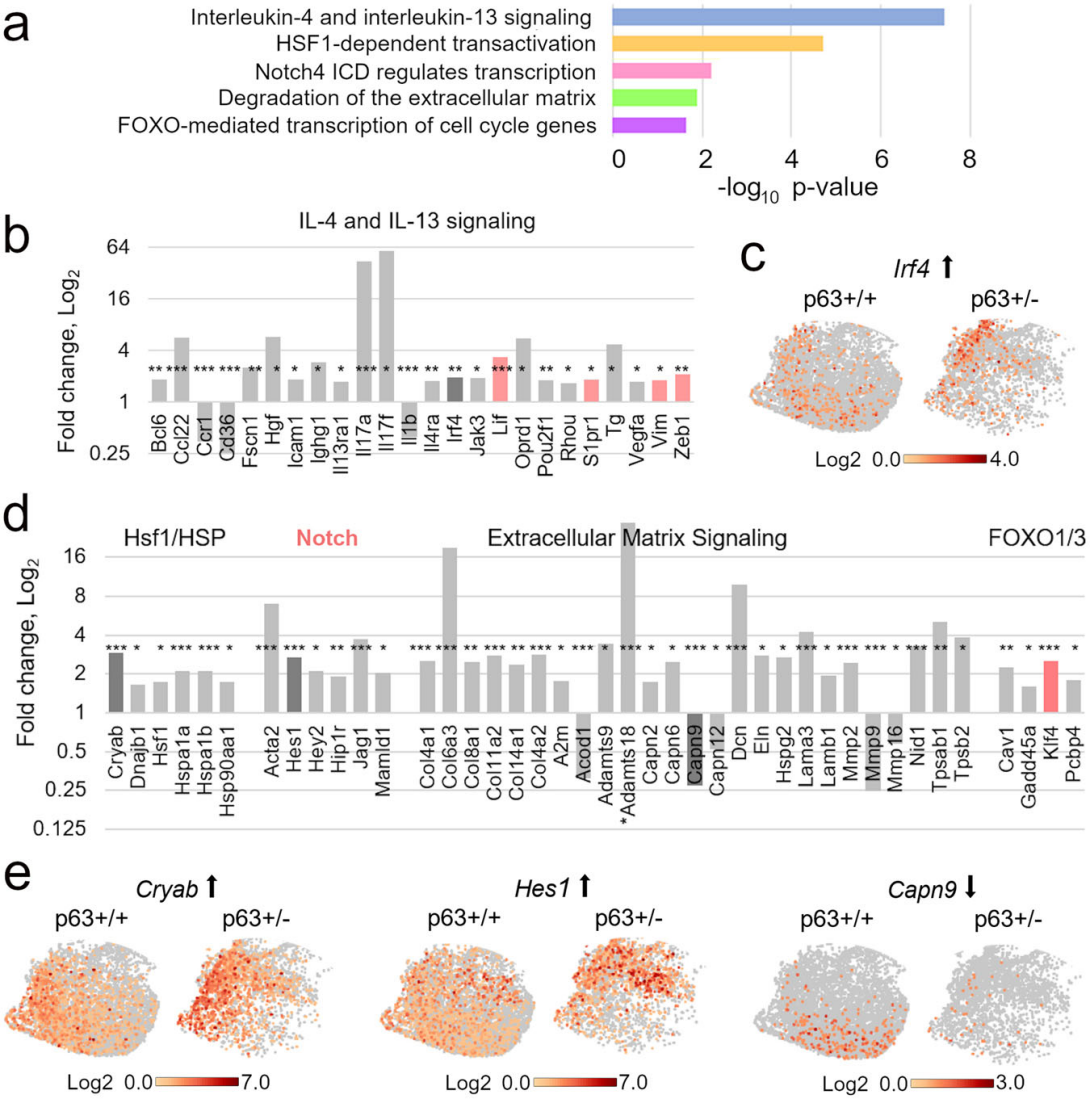
Pathways significantly altered in p63+/− vs. p63+/+ luminal mammary epithelial cells. **a** Representative pathways from the scRNA-seq analysis of the luminal p63+/− vs. p63+/+ clusters (defined as in Fig. 3b, c) revealed by the Reactome pathway analysis. See Supplementary Fig. 1 for the full list of enriched pathways. **b**–**e** Significantly altered genes in the individual pathways (**b**, **d**) and UMAP plots of selected genes (**c**, **e**). Dark gray bars, genes whose UMAP plots are shown; pink, stemness and self-renewal pathways and genes. *p < 0.05, **p < 0.01, ***p < 0.001.

**Fig. 5:**
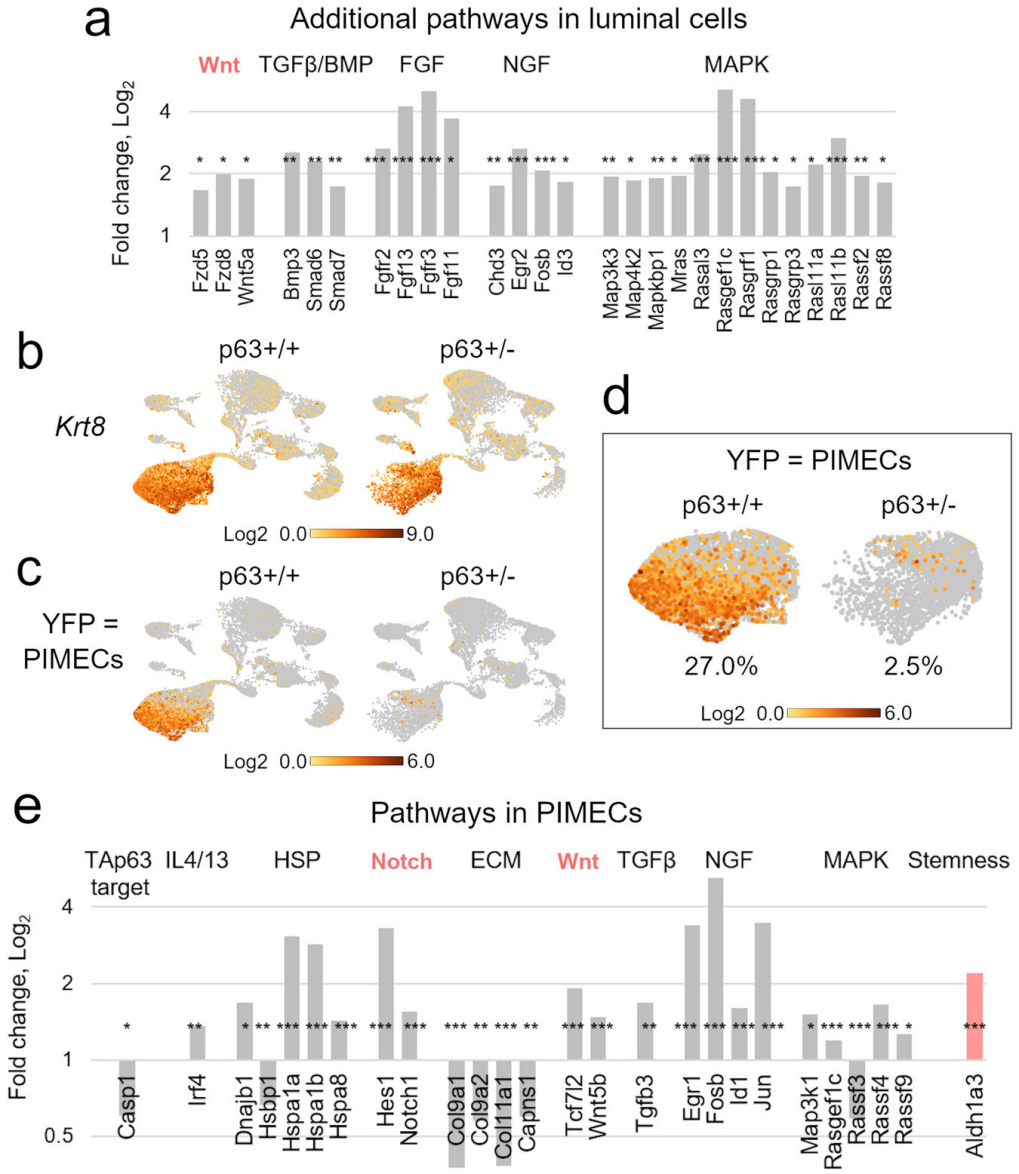
Additional pathways in the luminal cluster and analysis of p63+/− vs. p63+/+ PIMECs. **a** Additional pathways significantly altered in the p63+/− vs. p63+/+ luminal cluster (defined as in Fig. 3b, c) that were not revealed by Reactome. **b**–**e** Analysis of PIMECs, identified by *YFP/Krt8* double expression. **b**, **c** UMAP plots of re-aggregated clusters upon addition of the exogenous *YFP* gene, Loupe browser. Note slightly shifted cell positions compared to Fig. 3b, c, due to *YFP* addition and re-aggregation. **b** Luminal marker *Krt8* identifies the luminal cluster. **c** PIMECs are almost exclusively luminal cells, as expected. **d** A close-up of the luminal clusters. Note significant, >10-fold, decrease in the PIMECs number in p63+/− vs. p63+/+ MECs (**d**). **e** Pathways significantly altered in PIMECs closely resemble those altered in the luminal cells as a whole (**a**, also see Fig. 4). Pink, stemness and self-renewal pathways and genes. *p < 0.05, **p < 0.01, ***p < 0.001.

The uncovered role of p63 as a tumor suppressor in isolated PIMECs is in sharp contrast with our previous report that implicated p63 as an oncogene in ErbB2-overexpressing mammary glands^15^. The simplest explanation to unify these observations is that different assays reveal the roles of different p63 isoforms known to play opposite roles in cancer. Two major p63 isoforms are the full-length TAp63, a bona fide tumor suppressor similar to p53, and the N-terminally truncated ΔNp63, a bona fide oncogene^36–38^. We speculate that in intact p63+/− mice, ΔNp63—expressed exclusively in the basal cells^39–41^—maintains the number of post-pregnancy PIMECs, which are luminal cells^16,17^, in a non-cell-autonomous manner and thus, promotes HER2+ BC^15^ (see “Discussion”). On the other hand, TAp63—expressed exclusively in the luminal cells^39–41^ and therefore in PIMECs—cell-autonomously represses their tumorigenic properties, thus suppressing HER2+ BC (this study). To directly test this idea, first, we assessed the levels of *TAp63* and Δ*Np63* mRNAs by qRT-PCR in the luminal (CD24^high^) mammary cells in lieu of PIMECs, since very low PIMECs yields precluded their direct assessment by qPCR even upon combining mammary glands from several females (data not shown). We found that indeed, *TAp63* levels were somewhat reduced in p63+/− luminal cells (Fig. 3a, left). Surprisingly, we also found upregulation of *ΔNp63* in the luminal cells that normally is not expressed there (Fig. 3a, right). In order to gain deeper insight into the molecular underpinnings of p63+/− mammary gland, we then performed single-cell RNA sequencing (scRNA-seq) analysis on pooled CD24^pos^ (i.e., epithelial) mammary cells from p63+/+ (*n* = 4) and p63+/− (*n* = 3) mammary glands (Figs. 3b–e, 4, 5). Focusing on the PIMECs-enriched luminal cluster (Fig. 3b, orange), identified by the expression of *Krt8*, *Krt18*, *Epcam*, *Gata3* etc. (Fig. 3c, Supplementary Table 2), we assessed the expression of known TAp63 and ΔNp63 target genes. We found that among TAp63 targets, *Casp1* was significantly downregulated, while *Puma, Noxa, p21, Mdm2, Dicer* etc^37,42–45^ were not changed (Fig. 3d, e and data not shown). On the other hand, all significantly changed ΔNp63 targets were upregulated in p63+/− luminal cells (Fig. 3d, e). Subsequently, using more advanced software Seurat, we also found a significant downregulation of *Casp1* in p63+/− PIMECs per se (Fig. 5e, left). On a side note, our conventional scRNA-seq (from the 3′ end) did not directly detect *TAp63* and *ΔNp63* isoforms, likely because they are N-terminal. Altogether, these data are consistent with the idea that the increased tumorigenic capacity of p63+/− PIMECs is due to reduced TAp63 and possibly, induction of oncogenic ΔNp63 in the luminal cells.

### Upregulation of oncogenic and self-renewal pathways in p63+/− luminal cells and PIMECs

To further analyze p63+/− PIMECs at the molecular level, we then compared gene expression in p63+/− vs. p63+/+ luminal cells (in lieu of PIMECs) by the Reactome pathway enrichment analysis (www.reactome.org). We found that among significantly altered pathways were cancer-associated Il-4/Il-13^46^ and the Hsf1/HSP (heat shock proteins)^47^ pathways, the Notch pathway known to be induced by ΔNp63 and promote stemness^48,49^, the extracellular matrix (ECM) components known to promote cancer aggressiveness when overexpressed both, outside and inside of cancer cells^50,51^, and the FOXO pathway that can be both pro- and anti-oncogenic^52^ (Fig. 4, Supplementary Fig. 1). Interestingly, while most of the significantly altered genes were upregulated, some of the downregulated ones also signified an increased oncogenic capacity, e.g., *Capn9* (Fig. 4d, e), whose low level is a negative prognostic marker in breast cancer^53^. Furthermore, additional analysis of oncogenic signaling pathways revealed enrichment in Wnt (that mediates stemness downstream of ΔNp63), TGFβ, FGF, NGF, and MAPK pathways^28,51,54^ (Fig. 5a). Of note, several upregulated genes and pathways are associated with stemness and self-renewal, e.g., *Lif*, *Vim*, *Zeb1*, *Klf4*, the Notch and Wnt pathway (Figs. 4b, d, 5a, pink), thus explaining enhanced self-renewal of p63+/− PIMECs.

Using more advanced scRNA-seq analysis software, Seurat, we then zoomed in on the PIMEC cells (Fig. 5b–e). We found that the number of PIMECs was greatly reduced in p63+/−;ErbB2 compared to p63+/+;ErbB2 luminal cells (more than 10 folds, Fig. 5b–d), even more dramatically than we previously reported for non-ErbB2 glands (by 40%)^15^. Moreover, similarly to the overall luminal cells, p63+/− PIMECs were enriched in the cancer-associated and oncogenic Il-4/Il-13, HSP, TGFβ, FGF, NGF, and MAPK pathways^46,47,51,54^, the self-renewal Notch and Wnt pathways^28,48,49^, overexpressed a stemness marker *Aldh1a3*, and had significantly decreased TAp63 target gene, *Casp1* (Fig. 5e). The smaller number of significantly altered genes in each pathway is likely due to the smaller number of the PIMEC cells compared to the total luminal population, so that many genes did not reach statistical significance. Altogether, the scRNA-seq analysis revealed broad pro-oncogenic changes in p63+/− luminal cells in general and PIMECs per se, thus providing a mechanistic explanation for their increased tumorigenic capacity.

## Discussion

**Fig. 6:**
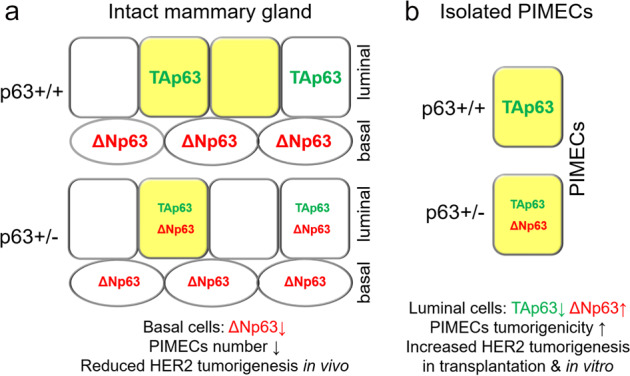
A model integrating the roles of p63 isoforms in PIMECs and pregnancy-associated HER2-positive breast cancer. **a** In the post-parous whole mammary gland, ΔNp63 (expressed in the basal cells) non-cell-autonomously maintains the post-pregnancy PIMEC pool, whereas TAp63 (expressed in select luminal cells) has minimal or no effect. This results in a decreased PIMEC number and suppressed HER2 tumorigenesis in p63+/−;ErbB2 females^15^. **b** In isolated wild-type PIMECs, the tumor suppressor TAp63 is the main isoform and suppresses their intrinsic tumorigenic properties, as well as keeps at bay oncogenic ΔNp63. In p63+/−;ErbB2 PIMECs, TAp63 is reduced, resulting in increased tumorigenic properties, due to ΔNp63 upregulation and activation of oncogenic and self-renewal pathways. Yellow, PIMECs.

Here we uncovered a previously unknown role of p63 (specifically, TAp63) as a cell-autonomous tumor suppressor in PIMECs that curbs their intrinsic tumorigenic properties in parous ErbB2 females. Together with our previous report of an oncogenic role of p63 towards the PIMECs content^15^ (likely due to oncogenic ΔNp63, which was not directly tested), this paints a more complete picture of a complex role of p63 in pregnancy-associated HER2+ BC (Fig. 6).

ΔNp63 is the major p63 isoform in stratified epithelia, since ΔNp63 knockout mice greatly phenocopy global p63 knockout, i.e., lack skin, mammary gland, prostate etc.^55^. ΔNp63 is also the predominant isoform in the mammary gland, where it is highly and exclusively expressed in the basal cells^39–41^. Clinically, ΔNp63 is frequently overexpressed in the bladder and squamous cell carcinomas^56–59^, where it acts as an oncogene by promoting tumor initiation, maintenance, and chemoresistance^60–63^. In breast cancer, p63/ΔNp63—along with CK5/14—is a histopathologic marker of the aggressive basal-like breast cancer^64,65^, and was proposed to contribute to poor outcomes in HER2+ BC as well. Indeed, patients with “HER2/basal” breast cancer have a significantly younger age of onset, shorter 5-year disease-free and overall survival, and increased metastasis^66–69^. “HER2/basal” tumors often are high-grade invasive ductal carcinomas^67^ or aggressive comedo-DCIS (ductal carcinoma in situ)^70^, and are innately resistant to Trastuzumab^67,69^. In addition, in the “HER2/basal” breast cancer patients with brain metastases, those metastases show even higher expression of both, HER2 and basal markers^71^. Mechanistically, ΔNp63 promotes carcinogenesis via dominant-negative effects against full-length family members (p53, TAp63, TAp73) and via independent transcriptional, anti-apoptotic, and anti-senescence mechanisms^25,36,37,60–62,72^. We previously proposed that ΔNp63 affects HER2+ BC in a non-cell-autonomous manner^15^ (Fig. 6a), especially since HER2+ BC is of luminal origin and ΔNp63 expression is normally basal^39–41^. In agreement, p63 depletion in the basal cells abolishes luminal milk-producing cells^41^, suggesting that basal ΔNp63 affects luminal PIMECs^16,17^ as the principal lobulo-alveolar progenitors^17,19^ (which was not directly tested). Here we show that ΔNp63 can also affect HER2+ BC cell-autonomously, by its aberrant upregulation in the luminal cells. This is consistent with the aforementioned “HER2/basal” BC phenotypes.

In contrast to ΔNp63, TAp63 is expressed in the mammary gland much weaker and exclusively in the luminal cells^39–41^. In breast and other cancers, TAp63 is a tumor suppressor, similarly to p53, and its expression correlates with patients’ positive outcomes and improved survival^44,59,65^. Mechanistically, TAp63 induces senescence or apoptosis of damaged cells, enhances chemosensitivity, and suppresses metastasis^37,43–45,73–75^. Loss of TAp63 in mouse models enhances tumorigenesis, via both p53-dependent and -independent mechanisms^73–75^. Moreover, loss of TAp63 drives aggressive metastatic mammary adenocarcinomas in mice, via upregulated Hippo pathway and accumulation of tumor-initiating and stem-like cells^43,76^. Consistently, we found that p63+/− PIMECs, that have decreased TAp63 and increased ΔNp63 function, show increased tumorigenic, self-renewal, and proliferative capacities (Fig. 6b) and overexpress numerous oncogenic signaling pathways (but not the Hippo pathway, data not shown).

Besides the N-terminal p63 isoforms, several alternatively spliced C-terminal isoforms (α, β, γ etc.) are known and affect p63’s activity^77^. However, we could not detect them by scRNA-seq, likely due to low expression and/or shared 3′ UTRs (data not shown). Moreover, it is unclear at present which C-terminal p63 isoforms are the most expressed and active in different MEC populations. Mechanistically, we found downregulation of *TAp63* in the luminal cells (Fig. 3a) and downregulation of its target gene *Casp1* in the luminal cells and PIMECs (Figs. 3d, e, and 5e, left), but no change in other known TAp63 targets. This is likely because different TAp63 target genes mediate its effects in different settings. Thus, TAp63 activates *Puma* and *Noxa* in the oocytes^78^. *p21*, usually activated upon DNA damage (which is not expected in the normal mammary gland), is mostly activated by TAp63β^79^, which is weakly expressed in the mammary gland^15^. And it is unclear whether *Mdm2* is activated by TAp63, ΔNp63 or both^80^. Nevertheless, our data reveal a novel role of p63, likely TAp63, as an essential tumor suppressor in pregnancy-associated HER2+ BC and its tumor-initiating cells PIMECs.

## Supplementary information

Supplementary material

## References

[1] Mitri Z, Constantine T, O’Regan R (2012). The HER2 receptor in breast cancer: pathophysiology, clinical use, and new advances in therapy. Chemother. Res. Pract..

[2] Eyermann CE, Haley JD, Alexandrova EM (2021). The HSP-RTK-Akt axis mediates acquired resistance to Ganetespib in HER2-positive breast cancer. Cell Death Dis..

[3] Miller KD (2004). The role of ErbB inhibitors in trastuzumab resistance. Oncologist.

[4] Nielsen DL, Kümler I, Palshof JA, Andersson M (2013). Efficacy of HER2-targeted therapy in metastatic breast cancer. Monoclonal antibodies and tyrosine kinase inhibitors. Breast.

[5] Elledge RM, Ciocca DR, Langone G, McGuire WL (1993). Estrogen receptor, progesterone receptor, and HER-2/neu protein in breast cancers from pregnant patients. Cancer.

[6] Shousha S (2000). Breast carcinoma presenting during or shortly after pregnancy and lactation. Arch. Pathol. Lab Med..

[7] Reed W, Sandstad B, Holm R, Nesland JM (2003). The prognostic impact of hormone receptors and c-erbB-2 in pregnancy-associated breast cancer and their correlation with BRCA1 and cell cycle modulators. Int J. Surg. Pathol..

[8] Middleton LP, Amin M, Gwyn K, Theriault R, Sahin A (2003). Breast carcinoma in pregnant women: assessment of clinicopathologic and immunohistochemical features. Cancer.

[9] Cruz GI (2013). Hypothesized role of pregnancy hormones on HER2+ breast tumor development. Breast Cancer Res. Treat..

[10] Cronin KA, Harlan LC, Dodd KW, Abrams JS, Ballard-Barbash R (2010). Population-based estimate of the prevalence of HER-2 positive breast cancer tumors for early stage patients in the US. Cancer Invest..

[11] Chen L (2016). Reproductive factors and risk of luminal, HER2-overexpressing, and triple-negative breast cancer among multiethnic women. Cancer Epidemiol. Biomark. Prev..

[12] Brouckaert O (2017). Reproductive profiles and risk of breast cancer subtypes: a multi-center case-only study. Breast Cancer Res..

[13] Henry MD, Triplett AA, Oh KB, Smith GH, Wagner KU (2004). Parity-induced mammary epithelial cells facilitate tumorigenesis in MMTV-neu transgenic mice. Oncogene.

[14] Haricharan S (2013). Mechanism and preclinical prevention of increased breast cancer risk caused by pregnancy. Elife.

[15] Yallowitz AR (2014). p63 is a prosurvival factor in the adult mammary gland during post-lactational involution, affecting PI-MECs and ErbB2 tumorigenesis. Cell Death Differ..

[16] Jeselsohn R (2010). Cyclin D1 kinase activity is required for the self-renewal of mammary stem and progenitor cells that are targets of MMTV-ErbB2 tumorigenesis. Cancer Cell.

[17] Chang TH (2014). New insights into lineage restriction of mammary gland epithelium using parity-identified mammary epithelial cells. Breast Cancer Res..

[18] Booth BW, Boulanger CA, Smith GH (2007). Alveolar progenitor cells develop in mouse mammary glands independent of pregnancy and lactation. J. Cell Physiol..

[19] Wagner KU (2002). An adjunct mammary epithelial cell population in parous females: its role in functional adaptation and tissue renewal. Development.

[20] Boulanger CA, Wagner KU, Smith GH (2005). Parity-induced mouse mammary epithelial cells are pluripotent, self-renewing and sensitive to TGF-beta1 expression. Oncogene.

[21] Matulka LA, Triplett AA, Wagner KU (2007). Parity-induced mammary epithelial cells are multipotent and express cell surface markers associated with stem cells. Dev. Biol..

[22] Mills AA (1999). p63 is a p53 homologue required for limb and epidermal morphogenesis. Nature.

[23] Yang A (1999). p63 is essential for regenerative proliferation in limb, craniofacial and epithelial development. Nature.

[24] Senoo M, Pinto F, Crum CP, McKeon F (2007). p63 Is essential for the proliferative potential of stem cells in stratified epithelia. Cell.

[25] Keyes WM (2011). ΔNp63α is an oncogene that targets chromatin remodeler Lsh to drive skin stem cell proliferation and tumorigenesis. Cell Stem Cell.

[26] Visvader JE, Lindeman GJ (2012). Cancer stem cells: current status and evolving complexities. Cell Stem Cell.

[27] Pignon JC (2013). p63-expressing cells are the stem cells of developing prostate, bladder, and colorectal epithelia. Proc. Natl Acad. Sci. USA.

[28] Chakrabarti R (2014). ΔNp63 promotes stem cell activity in mammary gland development and basal-like breast cancer by enhancing Fzd7 expression and Wnt signalling. Nat. Cell Biol..

[29] Muller WJ, Sinn E, Pattengale PK, Wallace R, Leder P (1988). Single-step induction of mammary adenocarcinoma in transgenic mice bearing the activated c-neu oncogene. Cell.

[30] Yallowitz A, Ghaleb A, Garcia L, Alexandrova EM, Marchenko N (2018). Heat shock factor 1 confers resistance to lapatinib in ERBB2-positive breast cancer cells. Cell Death Dis..

[31] Zheng GX (2017). Massively parallel digital transcriptional profiling of single cells. Nat. Commun..

[32] Booth BW, Boulanger CA, Anderson LH, Smith GH (2011). The normal mammary microenvironment suppresses the tumorigenic phenotype of mouse mammary tumor virus-neu-transformed mammary tumor cells. Oncogene.

[33] Wagner KU (1997). Cre-mediated gene deletion in the mammary gland. Nucleic Acids Res..

[34] Manuel Iglesias J (2013). Mammosphere formation in breast carcinoma cell lines depends upon expression of E-cadherin. PLoS ONE.

[35] Ward JM, Rehg JE (2014). Rodent immunohistochemistry: pitfalls and troubleshooting. Vet. Pathol..

[36] Wu G (2003). DeltaNp63alpha and TAp63alpha regulate transcription of genes with distinct biological functions in cancer and development. Cancer Res..

[37] Candi E (2007). TAp63 and DeltaNp63 in cancer and epidermal development. Cell Cycle.

[38] Chen Y (2018). A double dealing tale of p63: an oncogene or a tumor suppressor. Cell Mol. Life Sci..

[39] Nylander K (2002). Differential expression of p63 isoforms in normal tissues and neoplastic cells. J. Pathol..

[40] Li N (2008). Reciprocal intraepithelial interactions between TP63 and hedgehog signaling regulate quiescence and activation of progenitor elaboration by mammary stem cells. Stem Cells.

[41] Forster N (2014). Basal cell signaling by p63 controls luminal progenitor function and lactation via NRG1. Dev. Cell..

[42] Yang A (1998). p63, a p53 homolog at 3q27-29, encodes multiple products with transactivating, death-inducing, and dominant-negative activities. Mol. Cell.

[43] Su X (2010). TAp63 suppresses metastasis through coordinate regulation of Dicer and miRNAs. Nature.

[44] Celardo I (2013). Caspase-1 is a novel target of p63 in tumor suppression. Cell Death Dis..

[45] Gressner O (2005). TAp63alpha induces apoptosis by activating signaling via death receptors and mitochondria. Embo J..

[46] Fasoulakis Z, Kolios G, Papamanolis V, Kontomanolis EN (2018). Interleukins associated with breast cancer. Cureus.

[47] Saini J, Sharma PK (2018). Clinical, prognostic and therapeutic significance of heat shock proteins in cancer. Curr. Drug Targets.

[48] Du Z (2010). Overexpression of ΔNp63α induces a stem cell phenotype in MCF7 breast carcinoma cell line through the Notch pathway. Cancer Sci..

[49] Moriyama H (2018). Notch signaling enhances stemness by regulating metabolic pathways through modifying p53, NF-κB, and HIF-1α. Stem Cells Dev..

[50] Xu S (2019). The role of collagen in cancer: from bench to bedside. J. Transl. Med..

[51] Zarzynska JM (2014). Two faces of TGF-beta1 in breast cancer. Mediators Inflamm..

[52] Hornsveld M (2018). FOXO transcription factors both suppress and support breast cancer progression. Cancer Res..

[53] Davis J (2014). Low calpain-9 is associated with adverse disease-specific survival following endocrine therapy in breast cancer. BMC Cancer.

[54] Molloy NH, Read DE, Gorman AM (2011). Nerve growth factor in cancer cell death and survival. Cancers.

[55] Romano RA (2012). ΔNp63 knockout mice reveal its indispensable role as a master regulator of epithelial development and differentiation. Development.

[56] Wang TY (2001). Histologic and immunophenotypic classification of cervical carcinomas by expression of the p53 homologue p63: a study of 250 cases. Hum. Pathol..

[57] Hu H (2002). Elevated expression of p63 protein in human esophageal squamous cell carcinomas. Int. J. Cancer.

[58] Sniezek JC, Matheny KE, Westfall MD, Pietenpol JA (2004). Dominant negative p63 isoform expression in head and neck squamous cell carcinoma. Laryngoscope.

[59] Park BJ (2000). Frequent alteration of p63 expression in human primary bladder carcinomas. Cancer Res..

[60] Rocco JW, Leong CO, Kuperwasser N, DeYoung MP, Ellisen LW (2006). p63 mediates survival in squamous cell carcinoma by suppression of p73-dependent apoptosis. Cancer Cell.

[61] DeYoung MP (2006). Tumor-specific p73 up-regulation mediates p63 dependence in squamous cell carcinoma. Cancer Res..

[62] Ramsey MR, He L, Forster N, Ory B, Ellisen LW (2011). Physical association of HDAC1 and HDAC2 with p63 mediates transcriptional repression and tumor maintenance in squamous cell carcinoma. Cancer Res..

[63] Sen T (2011). DeltaNp63alpha confers tumor cell resistance to cisplatin through the AKT1 transcriptional regulation. Cancer Res..

[64] Ribeiro-Silva A (2005). p63 correlates with both BRCA1 and cytokeratin 5 in invasive breast carcinomas: further evidence for the pathogenesis of the basal phenotype of breast cancer. Histopathology.

[65] Coates PJ (2018). p63 isoforms in triple-negative breast cancer: ΔNp63 associates with the basal phenotype whereas TAp63 associates with androgen receptor, lack of BRCA mutation, PTEN and improved survival. Virchows Arch..

[66] Liu H (2008). Basal-HER2 phenotype shows poorer survival than basal-like phenotype in hormone receptor-negative invasive breast cancers. Hum. Pathol..

[67] Oliveras-Ferraros C (2010). Pathway-focused proteomic signatures in HER2-overexpressing breast cancer with a basal-like phenotype: new insights into de novo resistance to trastuzumab (Herceptin). Int J. Oncol..

[68] Bagaria SP (2012). Prognostic value of basal phenotype in HER2-overexpressing breast cancer. Ann. Surg. Oncol..

[69] Martin-Castillo B (2015). Cytokeratin 5/6 fingerprinting in HER2-positive tumors identifies a poor prognosis and trastuzumab-resistant basal-HER2 subtype of breast cancer. Oncotarget.

[70] Shekhar MP, Kato I, Nangia-Makker P, Tait L (2013). Comedo-DCIS is a precursor lesion for basal-like breast carcinoma: identification of a novel p63/Her2/neu expressing subgroup. Oncotarget.

[71] Shao MM (2011). A subset of breast cancer predisposes to brain metastasis. Med Mol. Morphol..

[72] Gallant-Behm CL, Espinosa JM (2013). ΔNp63α utilizes multiple mechanisms to repress transcription in squamous cell carcinoma cells. Cell Cycle.

[73] Flores ER (2005). Tumor predisposition in mice mutant for p63 and p73: evidence for broader tumor suppressor functions for the p53 family. Cancer Cell.

[74] Gunaratne PH (2019). Activating p53 family member TAp63: A novel therapeutic strategy for targeting p53-altered tumors. Cancer.

[75] Guo X (2009). TAp63 induces senescence and suppresses tumorigenesis in vivo. Nat. Cell Biol..

[76] Su X (2017). TAp63 suppresses mammary tumorigenesis through regulation of the Hippo pathway. Oncogene.

[77] Straub WE (2010). The C-terminus of p63 contains multiple regulatory elements with different functions. Cell Death Dis..

[78] Kerr JB (2012). DNA damage-induced primordial follicle oocyte apoptosis and loss of fertility require TAp63-mediated induction of Puma and Noxa. Mol. Cell.

[79] Helton ES, Zhang J, Chen X (2008). The proline-rich domain in p63 is necessary for the transcriptional and apoptosis-inducing activities of TAp63. Oncogene.

[80] Barton CE (2010). Novel p63 target genes involved in paracrine signaling and keratinocyte differentiation. Cell Death Dis..

