# Correction: p63 suppresses the ability of pregnancy-identified mammary epithelial cells (PIMECs) to drive HER2-positive breast cancer

**DOI:** 10.1038/s41419-021-03916-0

**Published:** 2021-06-30

**Authors:** Christopher E. Eyermann, Jinyu Li, Evguenia M. Alexandrova

**Affiliations:** Department of Pathology and Stony Brook Cancer Center, Stony Brook University, Stony Brook, NY 11794-8691 USA

**Keywords:** Breast cancer, Mechanisms of disease

Correction to: *Cell Death and Disease* 10.1038/s41419-021-03795-5, published online 22 May 2021

The original version of this article unfortunately contained a mistake in the title. The correct title should read “p63 suppresses the ability of pregnancy-identified mammary epithelial cells (PIMECs) to drive HER2-positive breast cancer”. The authors apologise for the error. The original article has been corrected.

