# Supplementary material for: p63 suppresses the ability of pregnancy-identified mammary epithelial cells (PIMECs) to drive HER2-positive breast cancer
Source: Cell Death Dis. 2021 May 22;12(6):525. doi: 10.1038/s41419-021-03795-5 (PMC8141055; doi:10.1038/s41419-021-03795-5)

**Supplementary Table 1.** Details of the mammosphere formation assays.

|  |  | Cells | Number of |  |  |  |  |
| --- | --- | --- | --- | --- | --- | --- | --- |
|  | ID | seeded | mammospheres | % MFE | Avg MFE |  |  |
| p63+/+;  ErbB2 | 707 | 8813 | 232 | 2.63 |  |  |  |
|  |  |  | 227 | 2.58 |  |  |  |
|  |  |  | 216 | 2.45 |  |  |  |
|  |  |  | 173 | 1.96 |  |  |  |
|  |  |  | 127 | 1.44 | 2.212539996 |  |  |
|  | 710 | 7158 | 171 | 2.39 |  |  |  |
|  |  |  | 128 | 1.79 |  |  |  |
|  |  |  | 135 | 1.89 | 2.02104871 |  |  |
|  | 599 | 7137 | 100 | 1.40 |  |  |  |
|  |  |  | 100 | 1.40 |  |  |  |
|  |  |  | 85 | 1.19 |  |  |  |
|  |  |  | 109 | 1.53 |  |  |  |
|  |  |  | 70 | 0.98 | 1.300302657 |  |  |
|  | 597 | 6547 | 109 | 1.66 |  |  |  |
|  |  |  | 95 | 1.45 |  |  |  |
|  |  |  | 96 | 1.47 |  |  |  |
|  |  |  | 60 | 0.92 |  |  |  |
|  |  |  | 31 | 0.47 | 1.194513182 |  |  |
|  | 600 | 10624 | 124 | 1.17 |  |  |  |
|  |  |  | 114 | 1.07 |  |  |  |
|  |  |  | 89 | 0.84 | 1.025978916 | Mean | SD |
|  | 508 | 17535 | 152 | 0.87 | 0.866837753 | 1.43687 | 0.550288 |
| p63+/-; | 727 | 7203 | 237 | 3.29 |  |  |  |
| ErbB2 |  |  | 273 | 3.79 |  |  |  |
|  |  |  | 205 | 2.85 |  |  |  |
|  |  |  | 158 | 2.19 | 3.029987505 |  |  |
|  | 547 | 7004 | 155 | 2.21 |  |  |  |
|  |  |  | 167 | 2.38 |  |  |  |
|  |  |  | 206 | 2.94 | 2.512730215 |  |  |
|  | 471 | 9667 | 285 | 2.95 |  |  |  |
|  |  |  | 284 | 2.94 |  |  |  |
|  |  |  | 228 | 2.36 |  |  |  |
|  |  |  | 186 | 1.92 |  |  |  |
|  |  |  | 113 | 1.17 |  |  |  |
|  |  |  | 121 | 1.25 | 2.098239686 |  |  |
|  | 523 | 14680 | 145 | 0.99 |  |  |  |
|  |  |  | 255 | 1.74 |  |  |  |
|  |  |  | 342 | 2.33 |  |  |  |
|  |  |  | 226 | 1.54 |  | Mean | SD |
|  |  |  | 187 | 1.27 | 1.573569482 | 2.303632 | 0.618194 |

**Supplementary Table 2**. Markers used to identify cell clusters in scRNA-seq analysis.

| **Cluster** | **Genes** | **Example** |
| --- | --- | --- |
| C1a. Luminal | CD24-hi, Csn3, Egfr, Epcam, Erbb2, Erbb3, Erbb4, Gata3, Krt8, Krt18 | 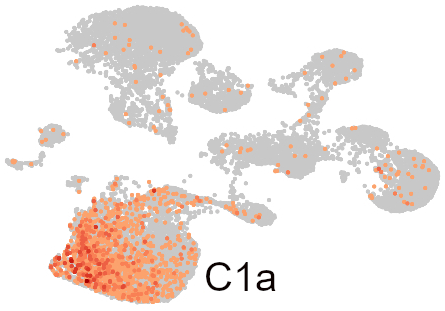Erbb2 |
| C1b. Basal | Acta2, Krt5, Krt14 | 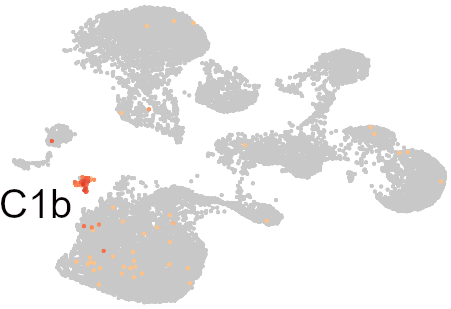 Krt14 |
| C2a. Naïve and memory B-cells | Bank1 (15x), Ebf1 (12x), Cd79a (12x), Cd79b (11x), Ms4a1 (11x), Igs  ([www.proteinatlas.org](http://www.proteinatlas.org)) | 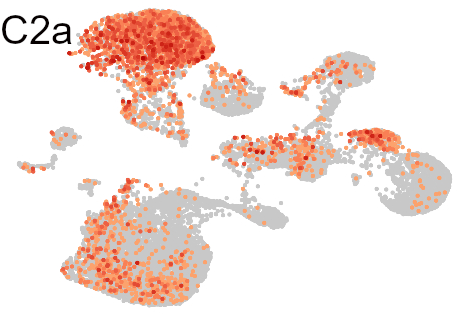Bank1 |
| C2b.T-cells | CD3e (132x), CD3g (178x), Cxcr6 (218x), TRBC1 (125x), TRBC2 (90x)  ([www.proteinatlas.org](http://www.proteinatlas.org)) | 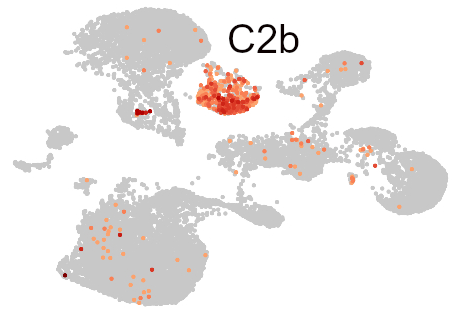CD3e |
| C3. Macrophages | Fpr1 (115x), Retnlg (147x), S100a8 (79x), S100a9 (87x), Trem1 (87x), Wfdc21 (87x)  ([www.proteinatlas.org](http://www.proteinatlas.org)) | 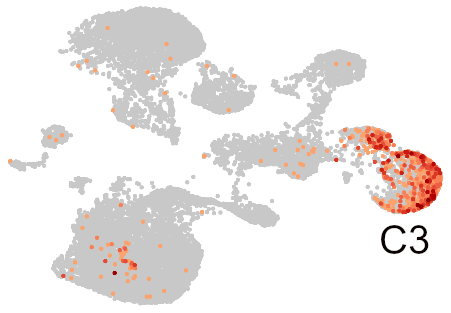 Fpr1 |
| C4. Stromal cells | S100a4*, Vim*, Pparg** | 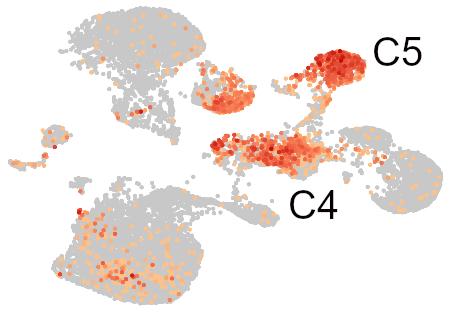S100a4 |
| C5. Stromal cells | S100a4*, Vim*, Tnfrsf9** | 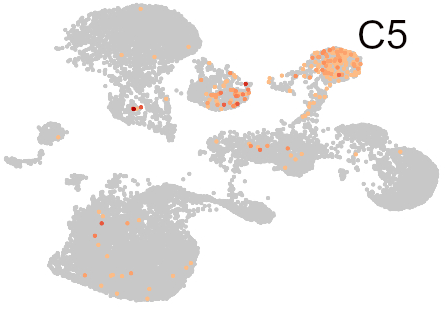Tnfrsf9 |
| C6a. B-cells | Igh (7-115x), Igk (15-323x), Igl (22-54x) | N/A*** |
| C6b. Red blood cells | Jchain (36x), Prg2 (108x)  ([www.proteinatlas.org](http://www.proteinatlas.org)) | 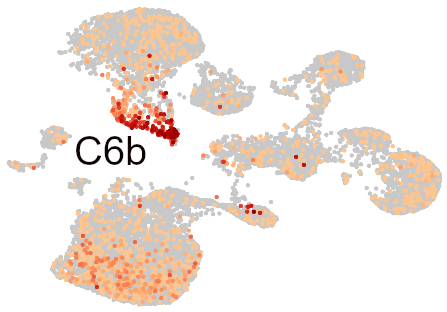Jchain |
| C7. Endothelial | Adgrl4 (1714x), Cdh5 (668x), Egfl7 (672x), Emcn (1442x), Fabp4 (621x), Kdr (811x), Ptprb (763x)  ([www.proteinatlas.org](http://www.proteinatlas.org)) | 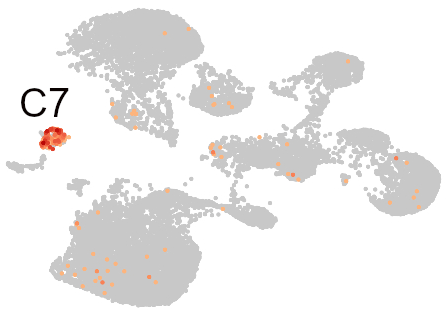Egfl7 |
| C8. Fibroblasts | Fbln1, Col1a1, Col1a2, Col5a1, Loxl1, Lum | 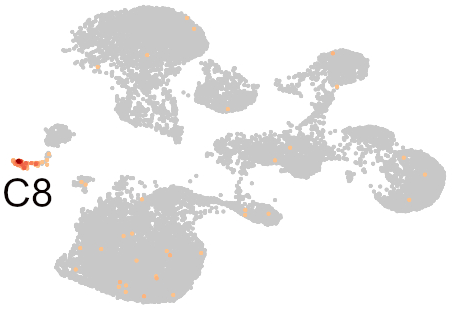 Col1a2 |

* Fibroblasts markers, ** adipocyte markers, *** no image shown due to very small number of individual

B-cells producing particular immunoglobulin variants, (Nx) indicates fold enrichment.

**Supplementary Figure 1**. Pathways significantly altered in p63+/-;ErbB2 compared to p63+/+;ErbB2 luminal cells. Significantly altered genes (p<0.05) were analyses by the Reactome overrepresentation analysis tool ([www.reactome.org](http://www.reactome.org)).


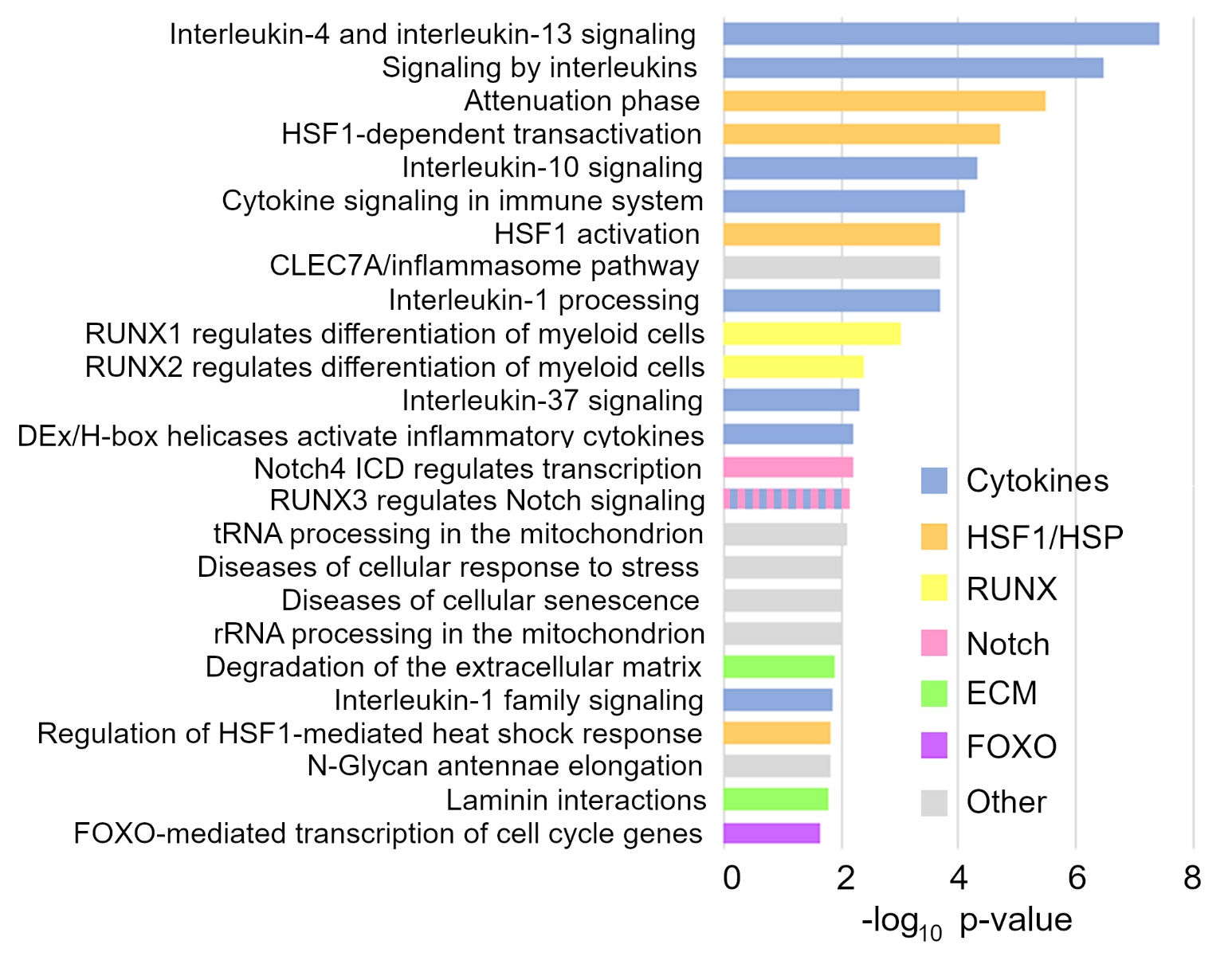

Supplement: Supplementary file 1 — Supplementary material [file 41419_2021_3795_MOESM1_ESM.docx]
